# Long Term Follow-Up on Pediatric Cases With Congenital Myasthenic Syndromes—A Retrospective Single Centre Cohort Study

**DOI:** 10.3389/fnhum.2020.560860

**Published:** 2020-12-07

**Authors:** Adela Della Marina, Eva Wibbeler, Angela Abicht, Heike Kölbel, Hanns Lochmüller, Andreas Roos, Ulrike Schara

**Affiliations:** ^1^Department of Neuropediatrics, Developmental Neurology and Social Pediatrics, University Children’s Hospital Essen, University Duisburg-Essen, Essen, Germany; ^2^Children’s Hospital, University Medical Center Hamburg Eppendorf, Hamburg, Germany; ^3^Medical Genetic Center Munich, Munich, Germany; ^4^Friedrich-Baur Institute, Ludwig Maximilian University, Munich, Germany; ^5^Children’s Hospital of Eastern Ontario Research Institute, Division of Neurology, Department of Medicine, The Ottawa Hospital, Brain and Mind Research Institute, University of Ottawa, Ottawa, ON, Canada; ^6^Department of Neuropediatrics and Muscle Disorders, Faculty of Medicine, Medical Center-University of Freiburg, Freiburg, Germany; ^7^Centro Nacional de Análisis Genómico (CNAG-CRG), Center for Genomic Regulation, Barcelona Institute of Science and Technology (BIST), Barcelona, Spain

**Keywords:** congenital myasthenic syndrome, neuromuscular transmission, therapy, long-term outcome, standardized testing

## Abstract

**Introduction**: Congenital myasthenic syndromes (CMS) refer to a heterogenic group of neuromuscular transmission disorders. CMS-subtypes are diverse regarding exercise intolerance and muscular weakness, varying from mild symptoms to life-limiting forms with neonatal onset. Long-term follow-up studies on disease progression and treatment-response in pediatric patients are rare.

**Patients and Methods**: We analyzed retrospective clinical and medication data in a cohort of 32 CMS-patients including the application of a standardized, not yet validated test (CMS-ST) to examine muscular strength and endurance in 21 patients at the last follow-up. Findings obtained in our cohort were compared with long-term follow-up studies of (adult) CMS-cohorts from the literature by considering the underlying molecular mechanisms. Outcomes of CMS-ST were compared to results of normal clinical assessment.

**Results**: Thirty-two pediatric patients with defects in eight different CMS-genes were followed by a median time of 12.8 years. Fifty-nine percentage of patients manifested with first symptoms as neonates, 35% as infants. While 53% of patients presented a reduced walking distance, 34% were wheelchair-bound. Even under adequate therapy with pyridostigmine (PS) and 3,4-diaminopyridine, *CHAT*-mutations led to the progression of muscular weakness partly in combination with persistent respiratory and bulbar symptoms. *RAPSN, CHRND*, and *CHRNB1* patients with neonatal manifestation, early respiratory problems, and bulbar symptoms showed a good and maintained treatment response. *CHAT* and *CHRNE* patients required higher PS dosages, whereas *RAPSN* patients needed a lower mean dosage at the last follow-up. The benefits of short-term medication and long-term progression of symptoms were highly dependent on the specific genetic defect. CMS-ST was carried out in 17/21 patients, determined affected muscle groups including bulbar and ocular symptoms, some of which were not reported by the patients.

**Conclusions**: Our findings and comparison with the literature- suggest a better treatment-response and less severe progression of symptoms present in patients suffering from mutations in CMS-genes directly associated with receptor deficiency, while patients with defects leading to synaptopathy and presynaptic defects tend to have worse outcomes. Assessment of affected muscular groups and clinical symptoms by CMS-ST may be a useful tool for optimal therapeutic management of the patients, especially for future clinical studies.

## Introduction

Congenital myasthenic syndromes (CMS) are a phenotypic heterogeneous group of hereditary diseases caused by disturbed or impaired neuromuscular transmission, resulting in clinical symptoms such as muscle weakness and increased muscle fatigue.

Clinical presentation and severity may vary enormously and depend on the underlying genetic mutation and the regular localization of the responding protein. Abnormal muscle fatigue can affect various muscle groups: limb-girdle, distal, proximal, cervical (axial), respiratory, eye, facial, and bulbar muscles. Notably, smooth and heart muscles are generally not affected (Abicht et al., 2017). The patients present their first symptoms usually at birth or in the first 2 years of life. Nevertheless, initial manifestations are known, albeit much less frequently, up to advanced age and then are frequently misdiagnosed as seronegative MG (especially mutations in the *RAPSN, DOK7*, and slow-channel syndrome; Croxen et al., 1997; Burke et al., 2003; Durmus et al., 2018; Kao et al., 2018).

In the recent decade, the genetic background of the CMS expanded, and currently, more than 35 causative genes have been published (Engel, 2018; Thompson et al., 2018; Vanhaesebrouck and Beeson, 2019). Remarkably, synaptic (*COLQ*) and postsynaptic mutations in acetylcholine receptor *(AChR), RAPSN*, and *DOK7* are more common than mutations in genes encoding for presynaptic localized proteins as exemplified on the cohort of the Mayo clinic where synaptic/postsynaptic defects account for 87% of the diagnosed cases (Engel, 2018).

The CMS subtypes are predominantly inherited in an autosomal recessive fashion, with exception of the slow-channel syndromes and the mutations in *SNAP25* and *SYT2*, which have an autosomal dominant inheritance (Engel et al., 2015; Abicht et al., 2017; Engel, 2018; Vanhaesebrouck and Beeson, 2019).

Long term follow-up studies in pediatric patients with CMS are rare and urgently needed for the counseling of the patients and their families. Up to now, there are no standardized tests in use in children for objective measurement of muscle strength and to assess the limits of the therapy.

To overcome both limitations, we retrospectively studied the clinical characteristics and medication data of 32 children with genetically proven forms of CMS. At their last follow-up visit, 21 patients had undergone a standardized, previously not validated test (CMS-ST) to examine their muscular strength and endurance. Patients were sub-grouped according to their genetic defect and associated pathomechanism and the main focus was spent on long-term problems and the limits of the drug therapy.

## Materials and Methods

At the University Hospital of Essen (Department for Neuropediatrics), a retrospective chart review was performed for all patients diagnosed with CMS who were at regular follow up between January 2010 and December 2015. Doing so, the medical records of 32 patients from 27 families were reviewed. The prerequisite for inclusion in this study was the genetically confirmed diagnosis of a CMS. The retrospective data were collected from the patient records. Written informed consent was obtained from the participants (or rather their legal guardians) for participation in the subsequent research as well as for the publication of the findings (including any potentially-identifying information). Ethical approval for this study was obtained from the University Essen clinical ethics committee (10-4543).

For this study, a questionnaire was used and completed together with the patients and their families, and a standardized clinical examination was carried out. The following data were obtained: sex, age, identification of a causative mutation, family history, course of birth and pregnancy, early childhood development, course of the disease, initial and current symptoms, age at the diagnosis, demographic background, and applied drug and supportive therapies. Treatment modalities, time, and duration of therapy were also recorded in all patients. For patients for whom either only retrospective data could be collected or for whom there were gaps during the survey, missing clinical data were extracted from the medical records.

To evaluate the current clinical status, in 18 patients older than 3 years willing to participate, the modified quantitative myasthenia gravis score (QMG) score was performed. The QMG score has been modified by us to allow assessments in children and infants with CMS and was applied at the last clinic visit (Chaouch et al., 2012). It is a standardized test (CMS-ST) procedure using two different forms depending on age. One is for infants and young children up to the age of three, the other for children from the age of three, adolescents, and adults (Supplementary Figures 1A,B).

The patients were categorized based on their genetic background in presynaptic, synaptic, and postsynaptic (with kinetic defects) sub-groups. Additionally, we compared our long-term data with clinical observations obtained in three independent long-term studies focusing on adult and mixed adult-pediatric CMS-cohorts (Natera-de Benito et al., 2017; Durmus et al., 2018; Kao et al., 2018).

### Statistical Analysis

Continuous variables (age, duration of medical treatment, length of the follow-up period) are reported as mean, minimum, and maximum values. Categorical variables (disease classification) are reported as frequencies and percentages based on the total sample.

## Results

### Patients and Demographics

The gender distribution in the present patient population was male to female 16:16.

Of 27 families, five families (19%) had two affected siblings, respectively. A total of 4 out of 27 families (15%) had one sibling who died in infancy or childhood. In 9/27 families (33%), the mother had at least one miscarriage. 6/27 families (22%) were consanguineous. For 12/27 families (44%) both parents came from Germany, for the remaining 15 families (56%) at least one parent was of non-German origin (Supplementary Table 1).

### Genetic Background and Patient Categorization

Given that a detailed classification of CMS disease entities is suitable for use in clinical and genetic databases and decision support systems (Thompson et al., 2018), our 32 patients were assigned to eight different genetic CMS sub-forms and further sub-categorized according to the location of the loss of the functional protein (based on the underlying genetic defect) at the NMJ (Supplementary Table 1): “Presynaptic mutations” (*CHAT*, OMIM: 254210) were found in five patients (16%), “synaptic mutations” (*COLQ*, OMIM: 603034) in three patients (9%) and “postsynaptic mutations” in a total of 24 patients (75%). Out of these 24 patients, 8 (25%) presented with pathogenic *RAPSN*-variants (OMIM: 616326), seven patients (22%) with *CHRNE* mutations (OMIM: 608931), three patients (9%) with *DOK7* mutations (OMIM: 254300), two patients (6%) with slow-channel *CHRNE* variants (OMIM: 605809), and each one patient (3%) with mutations in *MUSK* (OMIM: 616325), *CHRND* (OMIM: 616323), *CHRNB1* (OMIM: 616314), fast-channel *CHRNE* (OMIM: 616324), respectively.

### Age of Onset and Point of Diagnosis

Apart from categorization according to the underlying genetic defect and vulnerability at the NMJ, the patients were further classified according to the age of onset of their symptoms in three sub-groups: neonatal period, infancy, and older than 2 years.

**Figure 1:**
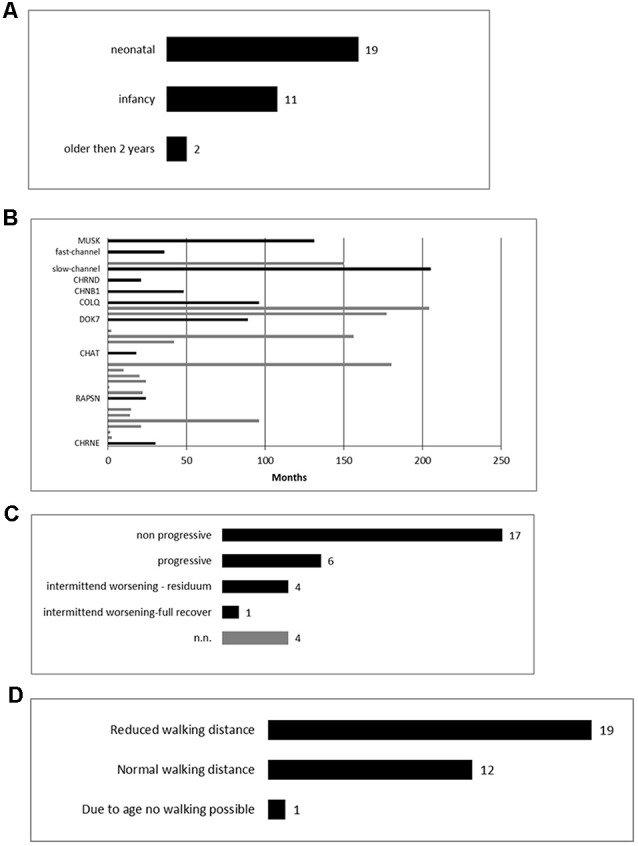
Clinical findings of our overall congenital myasthenic syndromes (CMS) cohort. **(A)** Age of clinical manifestation of the symptoms (*n* = 32). **(B)** Time from onset of the first symptoms to diagnosis (*n* = 29). **(C)** The clinical course of the patients (*n* = 32). **(D)** Walking distance at the point of clinical evaluation (*n* = 32).

The age onset varied from neonatal (59%) to 96 months (mean: 9.1 months; Figure 1A).

In 29 patients the exact age of diagnosis was stated with a mean of 69 months (range: 0–216 months). The mean duration between the onset of symptoms and diagnosis was 63.3 months (range 0–205 months; Figure 1B).

### Clinical Outcome

The mean follow-up period was 144, median 153 months (range 6–346 months) and although all patients improved during the pharmacological therapy for at least some of their symptoms, several still showed disease progression over time (Figure 1C).

### Motor Development and Endurance

Motor development in infancy was delayed in 19/32 patients (58%) and appeared to be regular in 13/32 patients (42%). In 29/32 patients (90%), intermittent worsening (respiratory worsening or/and increased muscular weakness) occurred during the disease.

The walking distance was limited in 18/32 patients (56%), in one patient (3%) walking was not yet possible due to her age (6 months, Figure 1D).

## Clinical Findings CMS-Patients Stratified by the Genetic Cause

### Presynaptic Mutations

#### Choline Acetyltransferase (ChAT) Deficiency

The presynaptic sub-cohort is defined by five patients with pathogenic *CHAT* mutations (Supplementary Table 1). Consanguinity of the parents was present in 3/4 families (two patients were siblings).

In 2/5 patients intrauterine reduced fetal movements were noticed and 3/5 patients showed first symptoms in the neonatal period. The mean time between the onset of symptoms and diagnosis was 3 years and 8 months (range: 0 months to 13 years), with one sibling being diagnosed directly neonatal. Motor development was normal in 3/5 patients. Clinical symptoms at onset are summarized in Table 1.

**Table 1:** Response to therapy, symptoms at onset, and long term course in patients according to the NMJ-localization of the protein affected by the respective mutation.

Classification	Gene	Symptom onset (number of pat)	Response to treatment (number of pat)	Clinical symptoms at onset	Long term course/problems
Presynaptic	*CHAT*	Neonatal (3/5) Infancy (2/5)	PS: improvement (5/5) DAP: additional improvement (2/5), no improvement (2/5)	Ptosis Facial and generalized muscular weakness Bulbar symptoms Intermittent worsening with respiratory insufficiency	Progressive muscular weakness Limited walking distance Scoliosis Bulbar symptoms Respiratory insufficiency with NIV Residual symptoms due to hypoxic injury
Synaptic	*COLQ*	Neonatal (2/3) Infancy (1/3)	PS: worsening (3/3) EPH: improvement (2/3)	Ptosis, ophthalmoparesis, Facial and generalized muscular weakness High arched palate Bulbar symptoms Intermittent worsening of symptoms with respiratory insufficiency Delayed motor development	Stabilization of respiratory problems Scoliosis Respiratory insufficiency Non-progressive course Limited walking distance
Postsynaptic	*CHRNE*	Neonatal (3/7) Infancy (4/7)	PS: improvement (7/7) DAP: additional improvement (3/7)	Ptosis, ophthalmoparesis Facial and proximal muscular weakness Delayed motor development	Stabilization of respiratory symptoms Limited walking distance No effect of therapy on ophthalmoparesis
	*CHRND*	Neonatal (1/1)	PS: improvement (1/1)	Ptosis, ophthalmoparesis Facial and generalized muscular weakness Bulbar symptoms Intermittent worsening of symptoms with respiratory insufficiency during infections Delayed motor development	Stabilization of respiratory symptoms Non-progressive course Joint contractures
	*CHRNB1*	Neonatal (1/1)	PS: improvement (1/1) DAP: additional improvement	Ptosis, ophthalmoparesis Facial and generalized muscular weakness Bulbar and respiratory symptoms Intermittent worsening with respiratory insufficiency	Stabilization of respiratory symptoms Ophthalmoparesis, facial muscular weakness Limited walking distance
Postsynaptic	*MUSK*	Neonatal (1/1)	PS: partial improvement (1/1) DAP: no effect (1/1) EPH: improvement (1/1)	Ptosis, ophthalmoparesis Facial and generalized muscular weakness, axial muscular weakness Bulbar and respiratory symptoms Intermittent worsening with respiratory insufficiency Delayed motor development	Stabilization of respiratory problems Persistent axial muscular weakness Limited walking distance Scoliosis Atrophic tongue
	*DOK7*	Infancy (2/3) Childhood (1/3)	PS: worsening (2/3), no effect (1/3) EPH: improvement (3/3)	Reduced fetal movements during pregnancy Ptosis, facial muscular weakness Intermittent worsening of symptoms with respiratory insufficiency Limb-girdle muscular weakness Delayed motor development	Intermittent worsening with partial improvement Improvement of respiratory symptoms Nasal speech Scoliosis Limited walking distance Muscular atrophy
	*RAPSN*	Neonatal (7/8) Infancy (1/8)	PS: improvement (8/8)	Reduced fetal movements during pregnancy Arthrogryposis multiplex congenita Ptosis, bulbar symptoms Respiratory insufficiency during infection Delayed motor development	Improvement of symptoms, non-progressive course Stabilization of respiratory symptoms Strabismus Scoliosis Joint contractures
Postsynaptic-kinetic	*CHRNE fast*	Neonatal (1/1)	PS: improvement (1/1) DAP: additional improvement (1/1)	Ptosis, ophthalmoparesis Facial and generalized muscle weakness Bulbar and respiratory symptoms Intermittent worsening of symptoms with respiratory insufficiency	Improvement of symptoms Limited walking distance
	*CHRNE slow*	Infancy (2/2)	PS: no effect (2/2) CHI: improvement (2/2)	Ptosis, ophthalmoparesis Muscular hypotonia in infancy Normal motor development Distal muscular weakness	Intermittent worsening with persistent weakness Limited walking distance

*Nr., number; pat, patients; PS, Pyridostigmine; DAP, 3,4 Diaminopyridine, EPH, Ephedrine, CHS, Chinidine sulfate; NIV, non-invasive ventilation*.

Notably, symptoms improved in all patients under therapy with pyridostigmine (PS), with a mean dosage of 5.0 mg/kg/day (range: 3.3–8.5 mg/kg/day). 2/5 patients were additionally treated with 3,4-diaminopyridine (3,4 DAP; mean dosage 1.1 mg/kg/day (range: 0.9–1.2 mg/kg/day) showing a further improvement in symptoms. Further 2/5 patients were treated with 3,4-DAP in the past without a notable effect. At their last follow-up, all patients had generalized muscular weakness and were wheelchair depended for longer distances. Moreover, 3/5 patients presented with intellectual disability (two of them had cerebral palsy as well as severe mental impairment as residuum due to hypoxic episodes during respiratory crises).

Clinical aspects of 2/5 patients were already published (Schmidt et al., 2003; Schara et al., 2010).

Based on the CMS-ST performed, in two patients, lateral diplopia could be provoked, muscular strength was severely reduced in proximal as well in distal muscles. Although respiratory insufficiency during infections was reported from both patients, FVC was normal (Supplementary Tables 2, 3).

### Synaptic Mutations

#### Endplate Acetylcholinesterase Deficiency (COLQ)

In three patients (from three independent families, consanguinity between the parents existed in 1/3 families, with one clinically affected father rejecting genetic testing) pathogenic mutations in the *COLQ* were identified (Supplementary Table 1).

In 2/3 patients, clinical onset was noticed in the neonatal period and in 1/3 patients during the first year of life. Only for one of these patients, the exact data concerning the diagnosis was available (at the age of 9).

The clinical symptoms at onset are summarized in Table 1.

The course of the disease was non-progressive in 2/3 of patients (one patient even had a normal walking distance). Two patients developed scoliosis and one was treated surgically.

All three patients experienced a worsening of their clinical symptoms under the therapy with PS. The therapy for all three was started before the genetic diagnosis was confirmed. Two patients were treated with ephedrine during the period of data collection and an improvement of their symptoms was noticed. The mean dosage was 4.4 mg/kg/day. In the case of one patient, the parents initially refused to administer ephedrine; thus she received the therapy later on and as a result, improved her walking distance. One patient showed an abnormal pupillary reflex.

Here, in only one patient the CMS-ST was performed. Her muscular strength in the upper extremities was poorer compared to lower extremities (Supplementary Table 2).

### Postsynaptic Mutations

#### CHRNE

Seven patients with pathogenic mutations in the *CHRNE* came from six independent families, three of them were consanguineous. For one patient, reduced fetal movements during pregnancy were reported. Ptosis and ophthalmoparesis were present and persisted in all patients (Table 1).

All patients were under therapy with PS. The mean dosage was 5.1-mg/kg/day (range: 1.3–11.9 mg/kg/day). Initially, clinical improvement was noticed in all of them, however, in 2/7 patients muscle weakness increased in the disease course despite medication. Furthermore, 3/7 patients additionally received 3,4-DAP at the time of their last follow-up visit (mean dosage was 1.4-mg/kg/day; range: 0.3–2.6 mg/kg/day).

6/7 patients experienced acute worsening of their symptoms partly triggered by infections.

At last follow-up, the walking distance was limited in 4/7 patients.

Based on the CMS-ST performed, in five patients a discrepancy between the possibility to step-up (possible in 5/5) and to perform the squats (possible in 3/5) was noticed. Muscular strength was more reduced in cervical muscles than in proximal and distal muscles (Supplementary Tables 2, 3).

#### RAPSN

In eight patients from six independent, non-consanguineous families pathogenic *RAPSN* mutations were identified; in 2/6 families, two children were affected in each case.

At neonatal presentation, 7/8 patients had a facial and generalized muscular weakness, and poor abilities to cry and suck, further complicated by respiratory problems (3/8 needed ventilatory support). All patients experienced intermittent episodes with worsening of their symptoms (in 4/8 muscular weakness and respiratory insufficiency), usually triggered by infections (Table 1). At the last follow-up, 3/8 patients reported chewing difficulties and in 2/8 joint contractions were noticed. One patient had moderate scoliosis which developed before PS therapy was initiated. Notably, 7/8 patients had normal walking distance. All patients improved under therapy with PS (mean dosage 3.3 mg/kg/day) and the course of the disease was non-progressive. Some of the clinical symptoms in two patients from this group have been already published (Schara et al., 2012).

Results of the standardized test applied in this group, showed in 2/6 patients reduced muscle endurance and in 3/6 reduced strength in neck flexors—both symptoms were not assessed through normal clinical assessment (Supplementary Tables 2, 3).

#### MUSK

A male patient from a non-consanguineous family with a pathogenic compound heterozygous *MUSK* mutation (Supplementary Table 1) was identified. The onset of the first symptoms was noticed in the neonatal period. He was tracheotomized from the age of 8 months to 8 years; due to respiratory insufficiency, he needed non-invasive ventilation during the night and partially during the day. He was able to walk at the age of 2 years and showed impaired mental development. His walking distance was limited due to proximal and axial muscular weakness (dropped head) and his muscular strength worsened during infections. Therapy with PS and 3,4-DAP was only partially effective as respiratory problems continued. After the confirmation of molecular genetic diagnosis at the age of 10 years, he received ephedrine and his respiratory situation, bulbar symptoms, and muscular strength improved.

#### CHRNB1

One female patient from a non-consanguineous family with CMS-causative mutations in the *CHRNB1* had the first onset of symptoms during the neonatal period. She experienced repeated episodes with worsening of her respiratory symptoms and muscular weakness up to the age of 2 years. She improved under therapy with PS (dosage 5.88 mg/kg/day), started at the age of 4 years. In addition, the patient received 3.4-DAP (0.44 mg/kg/day). Despite therapy, she experienced progression of her muscular endurance and at her last visit in our clinic (age 14 years and 6 months) presented with ptosis, ophthalmoparesis, facial and generalized muscular weakness accompanied by reduced walking distance.

#### CHRND

In one male patient from a non-consanguineous German family, compound heterozygous CMS-causative mutations in the *CHRND* were identified (Supplementary Table 1). His symptom onset was neonatal with a combination of facial, ocular, bulbar, and muscular symptoms. The patient improved under therapy with PS at the age of 21 months and had at last visit (age 13 years and 6 months) normal walking distance but distal finger joint contractures. The PS dosage was 5.2 mg/kg/day.

In CMS-ST, he had only mild reduced muscle endurance in his extremities, which was also reported during the normal clinical assessment (Supplementary Tables 2, 3).

#### DOK7

Two male and one female patient derived from three non-consanguineous families (all of German origin) with pathogenic *DOK7* mutations were identified in our CMS cohort (Supplementary Table 1). Clinical data of two patients have already been published (Schara et al., 2012; Shieh and Oh, 2018).

In two patients, the onset of symptoms (proximal weakness with reduced walking distance) was observed in infancy (12 months) and in one patient at the age of 6 years. The mean time between the onset of symptoms and diagnosis was 10 years 3 months (range: 7 years 5 months to 18). In one patient, the creatine kinase (CK) value was mildly increased (298 U/l).

Motor development in infancy was delayed in 2/3 of patients (axial weakness, in one generalized muscle weakness, and respiratory insufficiency with tracheostomy). All patients had ptosis and muscular hypotonia (limb-girdle muscle affected; in two patients generalized muscular atrophy was noticed).

Because of some clinical features suggestive of CMS, therapy with PS was given before the genetic diagnosis. A significant worsening of the condition was noticed in two patients; in one patient there was no effect upon therapeutic intervention. Although the diagnosis and therapy were considerably delayed, all three patients had marked improvement in their muscular endurance and better walking distance under therapy with ephedrine (average dose: 1.9-mg/kg/day; range: 0.8–3.6 mg/kg/day). One patient was even symptom-free. However, the patients also reported side effects under this therapy, including palpitations, dry mouth with a sore throat, increased perspiration, light intolerance due to mydriasis, and tremor.

One patient performed CMS-ST and showed extremely weak neck flexors in combination with proximal muscle weakness (no squats possible; Supplementary Table 2).

### Kinetic Defects of the Acetylcholine Receptor (AChR)

#### Slow-Channel CMS

In two female patients (siblings) of a non-consanguineous family (German origin; father also affected) pathogenic mutations leading to “slow-channel-CMS” were identified (Supplementary Table 1).

Muscular hypotonia as the first clinical symptom manifested at the age of 6 and 10 months but with normal further motor development. The course of the disease was progressive with episodes of muscular weakness; the younger sister had reduced walking distance. On clinical examination (aged 17 years and 11 months and 13 years and 15 months), both sisters had ptosis, ophthalmoparesis, and proximal and distal muscular weakness (especially hands and fingers).

Treatment with PS was discontinued in both due to lack of effect. The patients were under therapy with quinidine sulfate by mean dosage 6.6 mg/kg/day (range 6.1–7.2 mg/kg/day) at last follow-up. The older sister didn’t indicate any subjective improvement, the younger sister showed a clear improvement of her muscular endurance.

#### Fast-Channel CMS

In one male patient from a non-consanguineous family, a compound heterozygous mutation causative for the manifestation of “fast-channel-CMS” was identified (Supplementary Table 1). The onset of the symptoms was neonatal. His motor milestones were delayed, and he had respiratory crises during infancy.

At the age of 3 years and 9 months, therapy with PS was started leading to a partial improvement of the symptoms. Therefore, 3.4 DAP was added (PS 7.0 mg/kg/day and 3.4-DAP 1.1 mg/kg/day) with further benefit. Although under this therapy, there was no further progression of his weakness, muscular hypotonia, ptosis, and ophthalmoparesis persisted. His walking distance was moderately reduced.

In CMS-ST, he had reduced muscle endurance in his extremities, which was also reported during the normal clinical assessment. Due to his age, he was not able to perform all tasks (Supplementary Tables 2, 3).

### Standardized Testing (CMS-ST)

In three patients younger than 3 years, the standardized questionnaire was used (see Supplementary Figure 1A). Due to age, no standardized clinical endurance test was possible here. However, 18 patients were examined with the questionnaire for children from the age of 3 years, adolescents, and adults (Supplementary Figure 1B). Because of mental and motor impairment in one older patient (*CHAT* mutation, Supplementary Table 1, patient 1), no standardized clinical testing was possible.

A total of 14 out of the 17 patients underwent lung function testing (forced vital capacity; FVC): eight patients (*CHRNE*, *RAPSN*, and *CHRND*) presented with normal findings, whereas two (*CHRNE* and *RAPSN*) had mild and two (*RAPSN* and *COLQ*) had a moderate restrictive function, respectively. One *DOK7* patient even showed a severe restrictive ventilation disorder. There was no evidence of diaphragm weakness in lung function testing. Electrocardiogram (ECG) data were available for 15/18 patients and findings were all normal in all 15 cases. In patients between the age of 4 and 8 years, we observed only partial compliance in test performance (Supplementary Table 2). In Table 2, the results of testing in comparison to clinical assessments are presented showing more respiratory and bulbar symptoms identified by the test than reported by the patients themselves during the clinical assessment. Interestingly, in the testing of endurance, better performance was achieved by following the standardized investigation compared to the performance reported by the patients themselves (Table 2).

**Table 2:** Results of standardized test (CMS-ST) in 17/21 patients compared to neurological assessment and reported symptoms by patients and their parents.

Clinical symptom	CMS-ST (*n* = 17)	Clinical assessment and self-reported (*n* = 17)
Ocular symptoms	Ptosis: 15/17 patients, Double vision: 13/17 patients	Ptosis: 13/17 patients 9/17 double vision reported
Bulbar symptoms	10/17 patients	6/17 patients reported chewing problems
Respiratory function	6/14 abnormal	5/17 patients reported respiratory problems
Cervical weakness	15/17 patients	Not stated
Proximal weakness	10/17 patients	5/17 patients
Distal weakness	11/17 patients	Not stated
Impaired muscular endurance	8/17 Patienten	13/17 impaired muscular endurance reported

*Pulmonary function was performed in 14/17 patients; one patient was intellectually impaired and not capable to perform the test (total: *n* = 17)*.

## Discussion

Here, we present a cohort of pediatric patients with genetically proven CMS-causing mutations (Supplementary Table 1). Pathogenic variants affecting postsynaptic proteins were more prevalent (69% of patients), especially occurring in the *RAPSN* and *CHRNE* genes—known as frequent CMS-causative genes in Middle and Eastern European and North American cohorts as well as from single-center cohort from India (Abicht et al., 2012; Natera-de Benito et al., 2017; Engel, 2018; Selvam et al., 2018). Based on a relatively large proportion of patients with Turkish and Arabian background at our muscular center, *CHAT* mutations were more frequent compared to adult cohorts from Turkey and a reported mixed adult-pediatric cohort from Spain (Table 3A). In all patients, the phenotype at onset was identical with descriptions from the literature and may be used as a “clinical clue” toward starting of therapeutic intervention as long as the genetic testing is still pending (Abicht et al., 2012; Durmus et al., 2018; Engel, 2018). In the context of a comparison of our cohort with adult cohorts, it is important to note that for that purpose only patients with defects in genes also identified to be mutated in our cohort have been extracted from the different studies (Table 3A).

**Table 3A:** Comparison of findings in different CMS cohorts. Only patients with defects in genes also identified to be mutated in our cohort have been extracted from the different studies. Comparison of adult and mixed cohort patients and our cohort including follow-up time, point of symptom onset, time of diagnosis, and clinical symptoms depending on underlying mutation and characterization depending on the functional position on NMJ.

Study	Affected genes (Nr. of pat.)	Follow-up time (range)	Symptom onset (Nr. of pat.)	Onset to diagnosis	Long term problems in different CMS subtypes	Long term problems in pre-synaptic, synaptic, and post-synaptic forms
Natera-de Benito et al. (2017)	*CHAT* (2/64) *COLQ* (9/64) *CHRNE* (15/64) *DOK7* (7/64) *RAPSN* (11/64) Slow-channel (4/64)	Mean age at last follow up 27 years (range 0.5–80)	Neonatal (17/48) Infancy (20/48) Later (11/48)	Median time from onset to diagnosis: 7 years (range 0–47.5)	Ptosis: *CHAT, COLQ, CHRNE, RAPSN, DOK7*, slow-channel Ophthalmoparesis: *CHRNE*, slow-channel Bulbar symptoms: *CHAT, COLQ, CHRNE, RAPSN, DOK7*, slow-channel Proximal muscle weakness: *COLQ, CHRNE, RAPSN, DOK7*, slow-channel Distal muscular weakness: *CHAT*, slow-channel Cervical muscular weakness: *CHAT, COLQ, RAPSN, DOK7*, slow-channel Scoliosis: *COLQ, CHRNE, DOK7*, slow-channel Wheelchair dependence: *COLQ, CHRNE, DOK7*	Presynaptic: Ptosis, bulbar symptoms, distal and cervical muscle weakness Synaptic: Ptosis, bulbar symptoms, proximal and cervical muscular weakness, scoliosis, wheelchair dependence Postsynaptic: Ptosis, ophthalmoparesis, bulbar symptoms, proximal, distal, and cervical muscle weakness, scoliosis, wheelchair dependence
Durmus et al. (2018)	*CHAT* (1/69) *COLQ* (5/69) *CHRNE* (46/69) *DOK7* (3/69) Slow-channel (3/69) Fast-channel (3/69)	Median time: 9.8 years (1–22 years)	Neonatal (26/61) Infancy (13/61) Later: (15/61) (exact information was not provided for all patients)	Mean time from onset to diagnosis: 12.9 years	Ptosis: *CHAT, CHRNE*, slow-channel Ophthalmoparesis: *CHRNE*, slow-channel Bulbar symptoms: *COLQ* Proximal muscle weakness: *CHAT, COLQ, CHRNE, DOK7* Distal muscle weakness: COLQ, slow-channel Cervical muscle weakness: slow- channel Scoliosis: *COLQ* Wheelchair dependence: *CHAT* Ventilatory supp.: *CHAT, COLQ*, slow-channel	Presynaptic: Ptosis, proximal muscle weakness, ventilatory support Synaptic: Bulbar symptoms, scoliosis, ventilator support, proximal and distal muscular weakness Postsynaptic: Ptosis, ophthalmoparesis, proximal, distal, and cervical muscle weakness, ventilatory support
Kao et al. (2018)	*COLQ* (2/34) *CHRNE* (1/34) *DOK7* (14/34) *RAPSN* (6/34) slow channel (2/34)	Not stated	Neonatal and Infancy (11/34) Before 5 years of age (17/34) Later: (6/34)	Median time from symptom onset to diagnosis: 26.0 (4–56.5) years	Ptosis: *DOK7, RAPSN* Proximal muscle weakness: *COLQ, DOK7, RAPSN* Wheelchair dependence: *DOK7, COLQ* Ventilatory supp.: *RAPSN, COLQ*	Presynaptic: No patients Synaptic: Proximal muscle weakness, wheelchair dependence, ventilatory support Postsynaptic: Ptosis, proximal muscle weakness, wheelchair dependence, ventilatory supp.
Our cohort	*CHAT* (5/32) *COLQ* (3/32) *CHRNE* (7/32) *DOK7* (3/32) *RAPSN* (8/32) *MUSK* (1/32) Slow channel (2/32) Fast channel (1/32)	Mean time: 12 years (range 0.5–28 years)	Neonatal (19/32) Infancy (11/32) Older than 2 years (2/32)	Mean time from symptom onset to diagnosis: 5.3 years (range 0–17 years)	Ptosis: *CHAT, COLQ, CHRNE, DOK7, RAPSN*, MUSK, slow-channel Ophthalmoparesis: *COLQ, CHRNE, DOK7, MUSK*, slow-channel Bulbar symptoms: *CHAT, COLQ, CHRNE, RAPSN*, MUSK, fast channel Proximal muscle weakness: *CHAT, COLQ, RAPSN* Distal muscle weakness: *CHAT, COLQ, RAPSN* Cervical muscle weakness: *CHAT, COLQ, RAPSN* Scoliosis: *CHAT, COLQ, CHRNE, DOK7, RAPSN, MUSK* Wheelchair dependence: *CHAT, COLQ, CHRNE, DOK7* Ventilatory supp.: *CHAT, COLQ, DOK7, MUSK* Feeding tube: *CHAT, MUSK*	Presynaptic: Ptosis, bulbar symptoms (feeding tube), proximal, distal, and cervical muscular weakness, scoliosis, wheelchair dependence, ventilatory support Synaptic: Ptosis, ophthalmoparesis, bulbar symptoms, proximal, distal, and cervical muscular weakness, scoliosis, wheelchair dependence, ventilatory support Postsynaptic: Ptosis, ophthalmoparesis, bulbar symptoms, proximal, distal, and cervical muscular weakness, scoliosis, wheelchair dependence, ventilatory supp.

Interestingly, long term symptoms and problems show mostly similar findings with only some differences compared to the observations in two adult CMS- and one adult-pediatric cohort (Table 3A): in comparison to adults (34%), in our group, only 6% of patients had onset of symptoms after infancy. This discrepancy implies that our patients had an earlier onset and a more severe phenotype resulting in higher severity of symptoms in the long term. This observation might accord with the fact that for some of the CMS-subtypes discrepancy for clinical manifestation was reported, even in families with the same haplotypes (*CHAT*, *CHRNE*, *RAPSN*) (Burke et al., 2003; Schara et al., 2010; Natera-de Benito et al., 2016b). However, a bias can also be caused by retrospective memorizing of disease history when personal anamnesis is made in adult patients or based on a late consultation in adulthood although the disease showed first symptoms or even full clinical manifestation in childhood.

Nonetheless, an overall finding is that disease progression and response to therapies is highly gene- and pathomechanism specific in CMS as previously reported (Thompson et al., 2019).

### Presynaptic Mutations

Our data confirm the observation being made by Schara and colleagues (Schara et al., 2010) reporting that patients with presynaptic mutations in *CHAT* show progression in their muscular symptoms with persistent bulbar symptoms despite the application of standard therapy defining the need for targeted therapeutic strategies as recently described by Thompson and co-workers (Thompson et al., 2019). Worth noting, our findings support the concept of a beneficial effect of 3,4-DAP on muscular endurance as observed in the patients with *CHAT* mutations (see “Results” section), a therapeutic observation that was previously reported in only one child and one adult (Schara et al., 2010; Natera-de Benito et al., 2017).

Three patients developed scoliosis, for which no comparative data are available. Two out of three patients with scoliosis had residuum due to hypoxic episodes during infancy and are also mentally impaired. We postulate that the manifestation of scoliosis in these two patients occurred as the consequence of wheelchair dependency long-term in combination with severe axial and proximal weakness; one patient with preserved ambulation showed only mild scoliosis without the need for operative correction.

Although ocular symptoms are not known in *CHAT* patients, in ocular testing double vision could be provoked during longer glance to side as a symptom of ocular muscle weakness (Table 2, Supplementary Table 2). Cognitive impairment as a possible associated symptom have already been reported with partial association with hypoxic episodes during respiratory crises (Schara et al., 2010; McMacken et al., 2018). Based on an ubiquitous expression of ChAT, one might speculate that cognitive impairment observed in some patients might result from tissue-specific functions of the proteins which can (in some cases) only partially be compensated upon loss of the functional protein. However, compared to three adult patients described in adult cohorts (Natera-de Benito et al., 2017; Durmus et al., 2018), no information about their mental status was available.

### Synaptic Mutations

In the synaptic group, the symptoms can improve even after the late start of the therapy (patient 6) and most patients seem to have a non-progressive course of the disease. Still, the walking distance can be limited with wheelchair dependence for longer distances. Persistence of the symptoms in follow up is comparable with that found in adults (Durmus et al., 2018). Also similar as in adult cohorts, scoliosis can be one of the clinical symptoms specific to AChE deficiency, as already postulated (Mihaylova et al., 2008; Wargon et al., 2012; Duran et al., 2013; Durmus et al., 2018). A very specific indicator for this mutation is worsening of symptoms under therapy with AChE inhibitors (Wargon et al., 2012; Durmus et al., 2018), a clinical observation which could be confirmed in our sub-cohort. We could observe an abnormal pupillary reflex in only one patient-supporting previous reports describing the clinical observation as a less frequent feature (Mihaylova et al., 2008). Given that the presence of abnormal pupillary reflex is often considered as a “clinical clue” for AChE-deficiency patients, the absence of this finding does not exclude *COLQ* mutations as the causative genetic variant (Wargon et al., 2012). The patient of the synaptic sub-cohort show a combination of proximal and cervical muscular weakness whereas distal muscular weakness was not present in these patients (Table 3A) but reported in 8/15 patients of the Spanish adult cohort (Wargon et al., 2012).

### Postsynaptic Mutations

Compared to adults, long-term problems in pediatric cases of the postsynaptic group are similar and already present at the younger age, but improve over disease course: in case of *RAPSN* and *CHRNE* mutations, an improvement of neonatal symptoms such as bulbar problems or intermittent worsening as well in muscular endurance was observed and accord with known clinical observations described previously (McMacken et al., 2018). In our post-synaptic CMS cohort, a progression of muscular weakness under therapy could only be observed in singular cases suffering from *CHRNE* mutations. Although adult patients regularly improve under therapy, also here singular cases without optimal therapy response were reported in case of *CHRNE and DOK7* mutations (Natera-de Benito et al., 2016b; Durmus et al., 2018; Kao et al., 2018; Table 3B).

**Table 3B:** Response to applied therapy in two adult/one mixed and in our cohort.

Study	Pyridostigmine	3,4 Diaminopyridine	Acetazolamide	Albuterol/Salbutamole	Ephedrine	Fluoxetine	Chinidine
Natera-de Benito et al. (2017)	↑*CHRNE, RAPSN*	↑*CHRNE, RAPSN, DOK7*	-	↑*CHAT, COLQ, DOK7*, slow channel	↑*CHAT, COLQ, DOK7*, slow channel	slow channel	-
Durmus et al. (2018)	↑*CHAT, CHRNE*, slow channel(short), fast channel ↓ *DOK7*	-	-	↑*CHAT, DOK7*, slow channel	-	↑slow channel	-
Kao et al. (2018)	↑*CHRNE, DOK7, RAPSN* ↓*DOK7*, slow channel	↑*CHRNE, DOK7, RAPSN* ↓*DOK7*	↑ *RAPSN*	↑*COLQ, CHRNE, DOK7, RAPSN*	↑*DOK7, RAPSN*	↑slow channel	-
Our cohort	↑*CHAT, CHRNE, RAPSN*, fast channel ↓*COLQ, DOK7*	↑*CHAT, CHRNE*, fast channel	-	-	↑*COLQ, DOK7*	-	↑slow channel

*Nr., number; pat., patients; supp., support; ↑, improvement;↓, worsening*.

Persistent respiratory problems were present only in one *DOK7* and one *MUSK* patients, respectively. In this context, it is important to note that for both patients the start of an appropriate therapy was delayed (*DOK7* patient 18 years, *MUSK* patient 10 years) and both were already on ventilation before the ephedrine-therapy was started.

### General Therapy Response

Throughout the cohort presented here, drug therapy was started between the age of 3 weeks and 18 years. Whereas for pediatric cases no comparable long-term data are available from the present literature, those are reported for adults (Durmus et al., 2018 and Table 3A). Contrary to adults reporting the good effect of salbutamol/albuterol in patients with *CHAT, DOK7, COLQ, CHRNE, or RAPSN* mutations, we used ephedrine as monotherapy in case of *COLQ* and *DOK7* with good clinical response (Table 3B).

Although the cohort presented here is too small for an interpretation of the individual subgroups regarding the therapeutic effect, a progression of muscular weakness in a *CHAT* (five patients) group was observed despite therapy, indicating a less effective response to therapeutic intervention in this subgroup. If started administrated early, 3,4-DAP as we observed here, as well as of ephedrine/salbutamol already investigated in adult cohorts (Table 3B), might have an additional positive effect on long term outcome.

However, in siblings from the postsynaptic group (*RAPSN, CHRNE*) being treated very early, no severe worsening of the respiratory symptoms during the infections was observed. Thus, one might speculate that early therapeutic intervention in the postsynaptic group might prevent patients from worsening during infection triggered crises.

To our knowledge, this is the first CMS-cohort of children with mutations in genes encoding for different proteins localized to the NMJ where the dosage of therapy at the last follow-up visit has been calculated. Interestingly, only patients with *RAPSN* mutations need a low mean dosage of AChE inhibitors, at last follow up, to have a stable/static disease condition compared to patients with mutations in other CMS-causative genes. This not only has advantages based on a lower potential to suffer from side effects but also can be an indicator for stabilization of the symptoms in the course of the disease (Natera-de Benito et al., 2016a; Estephan et al., 2018). Interestingly, with 7.6 mg/kg/day the mean dosage administered to patients from a Spanish cohort (total of 10 patients, partially adults) was higher than in our patients (Natera-de Benito et al., 2016a). In our cohort the mean age at diagnosis and treatment start (3.4 years) was lower compared to the Spanish cohort (8.9 years)—so we postulate that early treatment in this group will reduce the need for medication later on.

In line with the known side effects of the drug at higher dosages, patients mentioned increased secretion production, diarrhea, and abdominal pain under the therapy with AChE inhibitors (Schara and Lochmüller, 2008).

As already described for adult CMS patients, in the case of the pediatric *DOK7*, *COLQ*, and “slow channel” patients included in this study, no beneficial changes occurred during the administration of AChE inhibitors or disease conditions even worsened significantly.

This therapy was started before the genetic diagnosis was confirmed due to some suggestive clinical features. In the case of *DOK7* patients, the weekly worsening of the muscular strength in combination with limb-girdle muscular weakness in the absence of ophthalmoplegia is the specific clinical clue. In the absence of abnormal pupillary reflexes, the phenotype of *COLQ* patients can overlap with other CMS with apnea (*CHRNE, CHRND, CHRNB1*) making the phenotypical differentiation difficult. Consequently, a very careful initiation of the therapy in case of negative genetic (or pending) findings is needed and the requirement of individual treatment strategies is suggested (Thompson et al., 2019).

### Standardized Test

Although our cohort was too small for standardized evaluation of the applied test, we could observe that younger patients (under the age of 10) have problems with the implementation especially due to lack of concentration, motivation, and compliance. Therefore, we conclude that ST-CMS (Supplementary Figure 1B) is not suitable for standard testing in patients under the age of 10 years, whereby normal cognitive development is also crucial, especially in the testing of the endurance of each muscle group (arm or leg holding).

Interestingly, performing CMS-ST disclosed more clinical symptoms than reported by the patients themselves (Table 2) and can differentiate the distribution of muscular weakness. Hence, this testing protocol may be one of the possible tools for a more precise assessment of the effect of therapeutic intervention in the case of prospective studies. Due to the differences in the phenotypical presentation in dependence of the underlying mutations in different CMS-causative genes, we would propose the use of the test as a tool for assessment of intra-individual effect of therapy.

## Data Availability Statement

The original contributions presented in the study are included in the article/Supplementary Materials, further inquiries can be directed to the corresponding author.

## Ethics Statement

The studies involving human participants were reviewed and approved by University Essen clinical ethics committee, 10-4543. Written informed consent to participate in this study was provided by the participants and their legal guardian/next of kin.

## Author Contributions

ADM designed the study and performed clinical follow up and study supervision as well as analysis and interpretation of data. ADM and AR designed the structure of the manuscript and wrote the first draft. EW performed clinical follow up studies, standardized testing and analysis and interpretation of data. HL provided the clinical questionnaire. AA provided the molecular genetic information. US designed the study and performed study supervision and critically revised the manuscript. EW, HK, HL and AA critically revised the manuscript and provided important intellectual content.

## Conflict of Interest

The authors declare that the research was conducted in the absence of any commercial or financial relationships that could be construed as a potential conflict of interest.

## Abbreviations

AChR: acetylcholine receptor
bw: body weight
ChAT: choline acetyltransferase
CMS: congenital myasthenic syndrome
CMS-ST: CMS standardized test
COLQ: endplate acetylcholinesterase deficiency
FVC: forced vital capacity
MG: Myasthenia gravis
NMJ: the neuromuscular junction
PS: Pyridostigmine
3,4 DAP: 3,4 Diaminopyridine.
