# Supplementary material for: Long Term Follow-Up on Pediatric Cases With Congenital Myasthenic Syndromes—A Retrospective Single Centre Cohort Study
Source: Front Hum Neurosci. 2020 Dec 7;14:560860. doi: 10.3389/fnhum.2020.560860 (PMC7750519; doi:10.3389/fnhum.2020.560860)
Supplement: Supplementary file 1 [file Data_Sheet_1.pdf]

Congenital Myasthenia Syndrome
Infant / Young Child Form (≤3 years)

1	**How is your baby / child currently fed?**	**Y**
	Breast Fed Bottle Fed Solids Drinks from cup Nasogastric tube/gastrostomy	
1a	**Breast / Bottle Details**	
	Does the baby splutter or choke during a milk feed?<br><br>How often? (Please circle) Every feed Once a day Once a week Other ……………………………………………………<br><br>What are the consequences of this choking / spluttering? (Please circle)<br><br>Recovers quickly stops feeding Respiratory distress Chest infections CI /frequent and requiring hospital admission How long does the baby take to complete a feed? (Millilitres per minute will give quantitative rate if bottle feeding)<br><br>……	☐<br><br>Minutes
1b	**Solids / Drinking Details**	
	Type of cup (Please circle) Open cup / beaker Beaker with lid Bottle other…………………………<br><br>Texture (Please circle) Eats all textures Eats cut up or small pieces Eats minced/ pureed food<br><br>……	
1c	**Details of nasogastric or gastrostomy feeds**	
	Undertaken because (Please circle) Top ups due to fatigue Safety/aspiration issues Other…………………………..<br><br>Comments ……	

2	**Is your child ventilated currently**	**Y**
	If yes give details ……	☐
3	**Crying**	**Y**
	Does your baby cry with a strong loud cry?	☐
	Does your baby cry with a weak quiet cry?	☐
	Does your baby's cry change whilst they are crying and if so how long after starting crying?	☐
4	**Talking or babbling**	**Y**
	Does your baby babble/talk?	☐
	Does their voice quality change with babbling/talking?	☐
	How long after they start babbling/talking?	
5	**Sleep**	**Y**
	How many sleeps per day does your child/baby have?	
	How long are the daytime sleeps?	
	How long is the night time sleep?	

6 Motor Milestones						
**Head Control**	Unable to maintain head upright	Wobbles	All the time maintained upright			
**Sitting**	Cannot sit	Sits with support at hips	Props	Stable sit	Pivots (rotates)	Observed:<br>Reported age:
**Voluntary grasp**	No grasp	Uses whole hand	Index finger and thumb but immature grasp	Pincer grasp		Observed:<br>Reported age:
**Ability to kick**	No kicking	Kicks legs horizontally / legs do not lift	Upward (vertically)	Touches leg	Touches toes	Observed:<br>Reported age:
**Rolling**	No rolling	Rolling to side	Prone to supine	Supine to prone		Observed:<br>Reported age:
**Crawling**	Does not lift head	Props on elbow normal at 3 months	Props on outstretched hand	Crawling flat on abdomen	Crawls on hands and knees	Observed:<br>Reported age:
**Standing**	Does not support weight	Supports weight	Stands with support	Stands unaided		Observed:<br>Reported age:
**Walking**		Bouncing	Cruising (walks holding on)	Walking independently		Observed:<br>Reported age:
**Score**	**0**	**1**	**1.5**	**2**	**Asymmetry**	**Comments**
**Facial Appearance**	Expressionless; does not react to stimuli	Closes eyes but not tightly; poor facial expression		Smiles or reacts to stimuli by closing eyes and grimacing		
**Eye appearance**	Continuous Deviation of eyes or abnormal movements	Intermittent Deviation of eyes or abnormal movements		Normal conjugated eye movement		
**Auditory response**	Does not react to stimuli	Doubtful reaction to stimuli or asymmetrical		Reacts to stimuli on both sides		
**Visual response**	Does not follow the arc	Follows the object for an incomplete arc, or asymmetry		Follows the object for a complete arc		

**Time started tests…………….**						
**Time of last medication…………… Medication:**						
**7 Timed Tests**						
	**Time in seconds**	**Change** ? ↑↓≈	**0**	**1**	**2**	**3**
**7a Ptosis**			>60	11-60	1-10	0
Asymmetry Y Details……						

**7b Ptosis**	Cannot open eyes at all	☐
	Can open eyes momentarily / briefly	☐
	Can open eyes for a few seconds	☐
	No ptosis	☐

7c	If they can sit independently how long is this for? (minutes)	
7d	If they need support how long can they tolerate before they need to change position? (minutes)	
7e	If they can lift their legs in the air how long can they do this for?	
7f	Props on extended forearms	
7c	If they can sit independently how long is this for? (minutes)	
7d	If they need support how long can they tolerate before they need to change position? (minutes)	
7e	If they can lift their legs in the air how long can they do this for?	
7f	Props on extended forearms	

Supplementary Figure 1A: standardized test for children up to 3 years.
