# Supplementary material for: Long Term Follow-Up on Pediatric Cases With Congenital Myasthenic Syndromes—A Retrospective Single Centre Cohort Study
Source: Front Hum Neurosci. 2020 Dec 7;14:560860. doi: 10.3389/fnhum.2020.560860 (PMC7750519; doi:10.3389/fnhum.2020.560860)
Supplement: Supplementary file 2 [file Data_Sheet_2.pdf]

**Congenital Myasthenia Syndrome**<br>**Child / Adult Form** (Appropriate for ≈ 3 years +)		
ECG<br>Details		

Factors influencing today's assessment		
	Fatigue<br>Last medication<br>Other (give details)	

1a	**Food Textures** Do you have to modify your food in any way in order to eat it?	**N/A**
	Eats all textures of food	**0**
	Eats cut up or small pieces of food or avoids hard/chewy foods	**1**
	Eats minced/ pureed food	**2**
	Minimal oral intake	**3**

1b	**Eating a meal** (with or without assistance) How long does it take to complete a whole meal?	**N/A**
	Able to consume a whole meal in the same time as others sharing the meal	**0**
	Able to consume a whole meal in the same time as others only with encouragement or needs some additional time (<10 min)	**1**
	Able to consume a whole meal but requires substantially more than 10 minutes extra compared to others eating the same meal or reduces portion size	**2**
	Unable to consume a whole meal even with additional time, assistance	**3**

1c	**Swallowing** Do you ever have problems with swallowing?	**N/A**
	Never has problems when swallowing and never chokes on food/drink,	**0**
	May experience occasional (less than once a month) problems swallowing certain types of food or occasionally chokes	**1**
	Has regular trouble swallowing food/drink or chokes on food/drink (more than once a month)	**2**
	Has trouble swallowing saliva or secretions	**3**

2 Respiratory

**Spirometry**	**sitting**	**lying**	
**FVC**			
**% predicted FVC**			

☐
☐
☐

Time started tests……………..
Time of last medication……………Medication:

3 ADAPTED QMG

See manual for different testing procedures for different ages

Timed Tests	Time in seconds	Change ? ↑↓≈	0	1	2	3
**Lateral Diplopia**			>60	11-60	1-10	0
**Ptosis**			>60	11-60	1-10	0
**Eyelid closure**			Normal	Some resistance	Unable to resist	Incomplete lid closure
**Head Lift to 45°**			>120	30-120	0-30	Unable
**Raise right arm to 90°**			>240	91-240	11-90	0-10
**Raise left arm to 90°**			>240	91-240	11-90	0-10
**Leg right raise to 45°**			>100	31-100	1-30	0
**Leg left raise to 45°**			>100	31-100	1-30	0
**Slurp Test**			Time it takes to drink 120ml of liquid from a beaker using a straw			
**Count to 50**			Time it takes to count to 50			
**“Me – Bee” Test**			Time it takes for a repetition of to become unclear			
	**Number**					
**Step-ups**			Number in 1 minute (use same step) height =....................cm			
**Squats**			Number in 1 minute			

4 Myometry

Test Dominant side only unless recognized asymmetry. Dominant R L

☐ Kilograms<br>☐ Newtons<br>☐ Pounds	**Right**		**Left**	
	**Muscle / Muscle Group**	**Result (Maximum Force)**	**Muscle / Muscle Group**	**Result (Maximum Force)**
Grip strength	…………….<br>……………<br>…………….	…………….	…………….<br>……………<br>…………….	…………….
Shoulder flexors	…………….<br>……………<br>…………….	…………….	…………….<br>……………<br>…………….	…………….
Knee extensors	…………….<br>……………<br>…………….	…………….	…………….<br>……………<br>…………….	…………….
Neck flexors	…………….<br>……………<br>…………….	…………….		
Grip endurance	Maximum grip pre repetitions	Repetitions done using	Reps done	Maximum grip strength post repetitions
	…………….	…………….	10<br>Other ……….	…………….

**5 Manual Muscle Testing (MMC)**				
Shoulder flexors				
Knee extensors				
Neck flexors				

Supplementary Figure1B. Standardized testing for children and adults older than 3 years.
